# Jerzy Jurka: June 4, 1950 – July 19, 2014

**DOI:** 10.1186/s13100-014-0032-2

**Published:** 2015-01-14

**Authors:** Elzbieta Jurka

**Affiliations:** Genetic Information Research Institute, 5150 El Camino Real, Ste B-30, Los Altos, CA 94022 USA

Jerzy Jurka, biologist, pioneer in the field of mobile DNA, and the founder, President and Director of Research at Genetic Information Research Institute (GIRI) from 1994 to 2014, died on July 19 at his home in Los Altos, California. He was 64.

**Figure 1:**
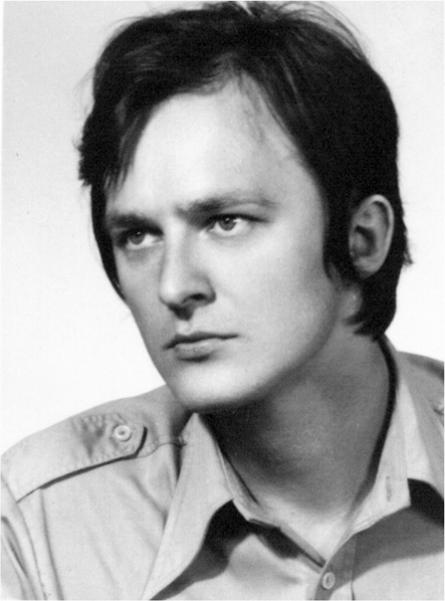
**Jerzy Jurka 1950-2014.**

Jerzy was born on June 4, 1950 in the small village of Ponikiew in Poland and grew up outside the town of Wadowice. His father, a teacher, influenced Jerzy’s interest in biology. In 1968, after finishing high school in Wadowice, Jerzy entered the Jagiellonian University in Krakow. He received a master’s degree in chemistry from the Jagiellonian University in 1973. Subsequently, in 1974, Jerzy accepted a position in the laboratory of Prof. Mieczyslaw Chorazy at the Maria Sklodowska-Curie Institute of Oncology in Gliwice. While in Gliwice, Jerzy divided his time between carrying out research and studying for his doctorate (Figure 1). His first paper titled “On replication of nucleic acids in relation to the evolution of genetic code and of proteins” was published in 1977. Two years later, he received his doctoral degree in molecular biology from the University of Warsaw in 1979. During this time, Jerzy sought opportunities beyond the Iron Curtain, accepting invitations as a visiting scientist in the laboratory of Prof. Dr. Manfred Eigen, a Nobel Laureate in Chemistry, at the Max Planck Institute in Gottingen, Germany and in the laboratory of Dr. Chapelville at the Centre National de la Recherche Scientifique (CNRS) in Paris, France, where he worked with Dr. Jacques Ninio. In 1982, acting on a joint invitation from Dr. Herrick Baltscheffsky of the Arrhenius Laboratory at Stockholm University and Dr. Rudolf Rigler of the Karolinska Institute, Jerzy left for Sweden shortly after martial law was imposed in Poland.

Jerzy immigrated to the United States with his wife Elzbieta in 1983. After serving as a postdoctoral researcher at the University of Michigan with Dr. Michael Savageau, and later at the University of Houston with Dr. George Fox, he worked as a research fellow at the Dana-Farber Cancer Institute Harvard School of Public Health in the laboratory of Dr. Temple Smith. There, he published his seminal article “A fundamental division in the Alu family of repeated sequences.” Alu elements and other transposable DNA elements would become the focus of Jerzy’s research over the course of the following years. At the same time, together with Don Faulkner, Jerzy designed and implemented an editor for genetic sequences called Multiple Aligned Sequence Editor (MASE) to simplify the manipulation of aligned sequence sets. Jerzy eventually made his way to California where in 1987 he accepted a position at Bionet, one of the earliest bioinformatics community projects on the Internet, which was started as part of the GenBank public biosequence data project by Intelligenetics at Stanford University. Later, after meeting Dr. Emile Zuckerkandl, he joined the Linus Pauling Institute of Science and Medicine in Palo Alto, California in 1989, and served as the Assistant Director of Research for Linus Pauling from 1992 to 1994.

**Figure 2:**
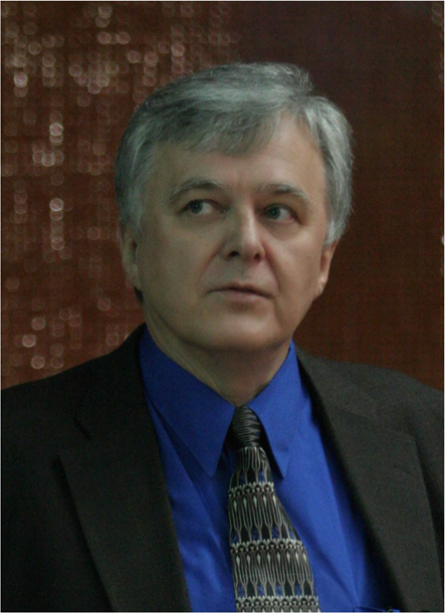
**Jerzy Jurka 1950-2014.**

Following the death of Pauling, Jerzy founded Genetic Information Research Institute (GIRI) in 1994 after securing initial funding from the U.S. Department of Energy Human Genome Program and the National Institutes of Health, National Library of Medicine. For more than two decades, he tirelessly built and maintained Repbase, the fundamental database of mobile DNA elements, and continued to publish original research on newly discovered mobile DNA elements, their mechanisms, and their evolution. He authored more than 120 scientific papers, as well as book chapters and encyclopedia articles on repetitive DNA elements. *Repbase Update*, developed under Jerzy’s direction since 1990, has become a pivotal resource for the mobile DNA research community and has been used in genome sequencing projects worldwide as a reference collection for the masking and annotation of repetitive DNA. In 2001 Jerzy established the electronic journal *Repbase Reports*, with the goal of providing the research community with a rapid publication devoted to organizing, studying, and permanently recording the diverse and rapidly growing information on repetitive DNA. Jerzy’s passion and enthusiasm inspired many younger scientists to embark on careers to study mobile DNA, and he helped foster the mobile DNA research community by organizing three international conferences at the Asilomar Conference Grounds on Monterey Peninsula in California (Figure 2). Over the course of more than two decades, Jerzy served as a member of the editorial boards for a number of scientific journals including *Journal of Molecular Evolution* (member of the Editorial Board 1989–1998; Associate Editor 1998–2006), *Biology Direct*, *Gene*, and *Mobile DNA*, and presented numerous invited lectures at scientific meetings and universities around the world.

Jerzy was known for his generosity, and he helped countless young academics start their careers, many of them immigrants like himself. He was a passionate amateur historian and was known to energize any discussion about history, science, or religion. He was also an avid chess player and connoisseur of music. Most of all, Jerzy was a loving husband and father to three sons, Michael, Matthew, and Timothy.

A Requiem Mass was held on July 26, 2014 at St. Simon Catholic Church in Los Altos, CA, with Prof. William Mahrt leading the St. Ann choir in Gregorian chants.

*This obituary was originally posted on the GIRI website (*http://www.girinst.org/about/jjurkabiosketch.html*) and has been republished in Mobile DNA by permission of Elzbieta Jurka.*

